# Supplementary material for: Health Impact of Air Pollution from Shipping in the Baltic Sea: Effects of Different Spatial Resolutions in Sweden
Source: Int J Environ Res Public Health. 2020 Oct 29;17(21):7963. doi: 10.3390/ijerph17217963 (PMC7663031; doi:10.3390/ijerph17217963)
Supplement: Supplementary file 1 [file ijerph-17-07963-s001.pdf]

International Journal of
Environmental Research and Public Health

MDPI

Supplementary Information

2.4. Exposure-response function and mortality calculation

Premature mortality was calculated as a linear function of the exposure response function:

$$Y = (RR-1)/RR \times B \times 0.95 \qquad (1)$$

Where Y is the number of extra premature deaths,

RR-1/RR is the population attributable fraction (PAF),

B the baseline mortality rate.

Years of life lost (YLL) calculations used the following equation:

$$Y_{county} = (Y^*n^*e)/(N^*E) \qquad (2)$$

Where Y is YLL for all Sweden,

n is the number of people living in the county,

e is the average exposure (μg/m^3) for the year,

N is the population of Sweden as a whole,

E is the average population exposure (μg/m^3) in per county Sweden for the year

2.6. Morbidity calculations

According to the World Health Organization, the burden of disease in terms of DALY in the general population is expressed as the equation

$$DALY = YLL + YLD \qquad (3)$$ [1]

Where YLL includes all of Sweden,

YLD is years lost due to disability.

To estimate the number of productive years lost due to the disease

$$YLD = I \times DW \times D \qquad (4)$$

Where, I = number of incident cases,

DW = disability weight. DW is associated with each health condition and lies on a scale of 0 (indicating optimal health) to 1 (indicating a condition equivalent to death). For the purpose of this study the DW used for MI and stroke were obtained from Salomon et al. [2].

D = average duration of the case until remission or death (years). The average duration for stroke and MI was obtained from Eriksson et al. [3].

Table 1. Summary of Baltic Sea ship and for the smaller domain around Gothenburg area emissions during 2014 and 2016.

Baltic Sea	Main_Fuel	AUX_Fuel	NOx *	SOx **	$PM_{2.5}$	CO	CO_2	Travel	Transport Work	VOC	SHIPS
	[10^3 tonnes]	[10^3 tonnes]	[tonnes]	[tonnes]	[tonnes]	[tonnes]	[10^3 tonnes]	[10^3 km]	[10^6 tonne km]	[tonnes]	
**Baltic Sea, 2014**	3705	1025	312946	75148	15002	4870	14380	137963	966574	2554	20159
**Baltic Sea, 2016**	3635	1167	315893	9727	9390	4881	14594	139205	998660	2612	22575
**Gothenburg, 2014**	121	58	11998	2579	540	826	545	5370	31800	96	8507
**Gothenburg, 2016**	130	69	13226	404	388	932	604	6007	35290	107	9328

*NOx calculated as NO_2; **SOx calculated as SO_2.

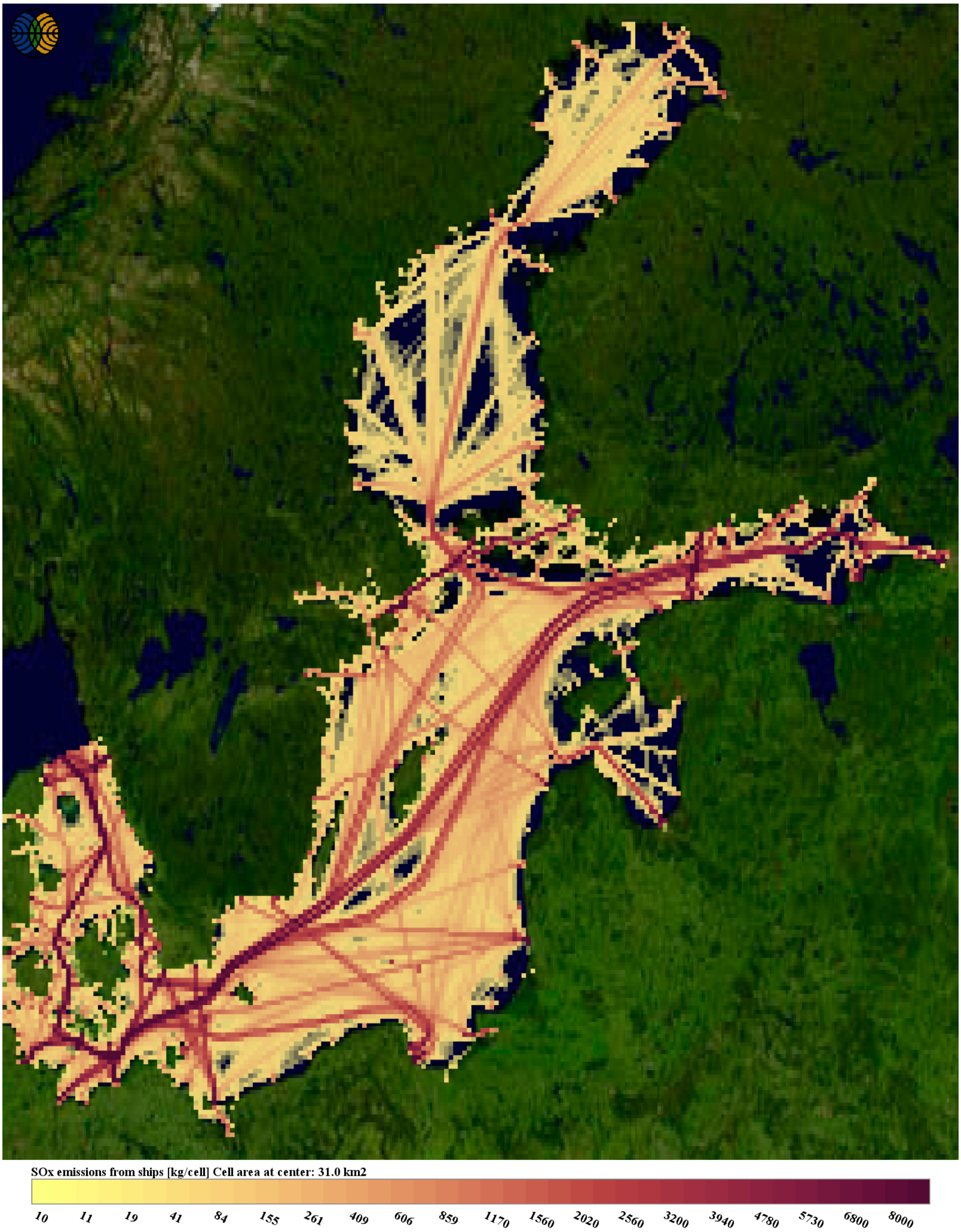

Figure S1. Emissions of SOx from Baltic Sea shipping in 2016. The unit of emissions is kg per grid cell area. Total SOx emissions during 2016 were 9727 tonnes, down from 75148 tonnes during 2014. This reduction was because of 0.1%S required in the Baltic Sea SECA.

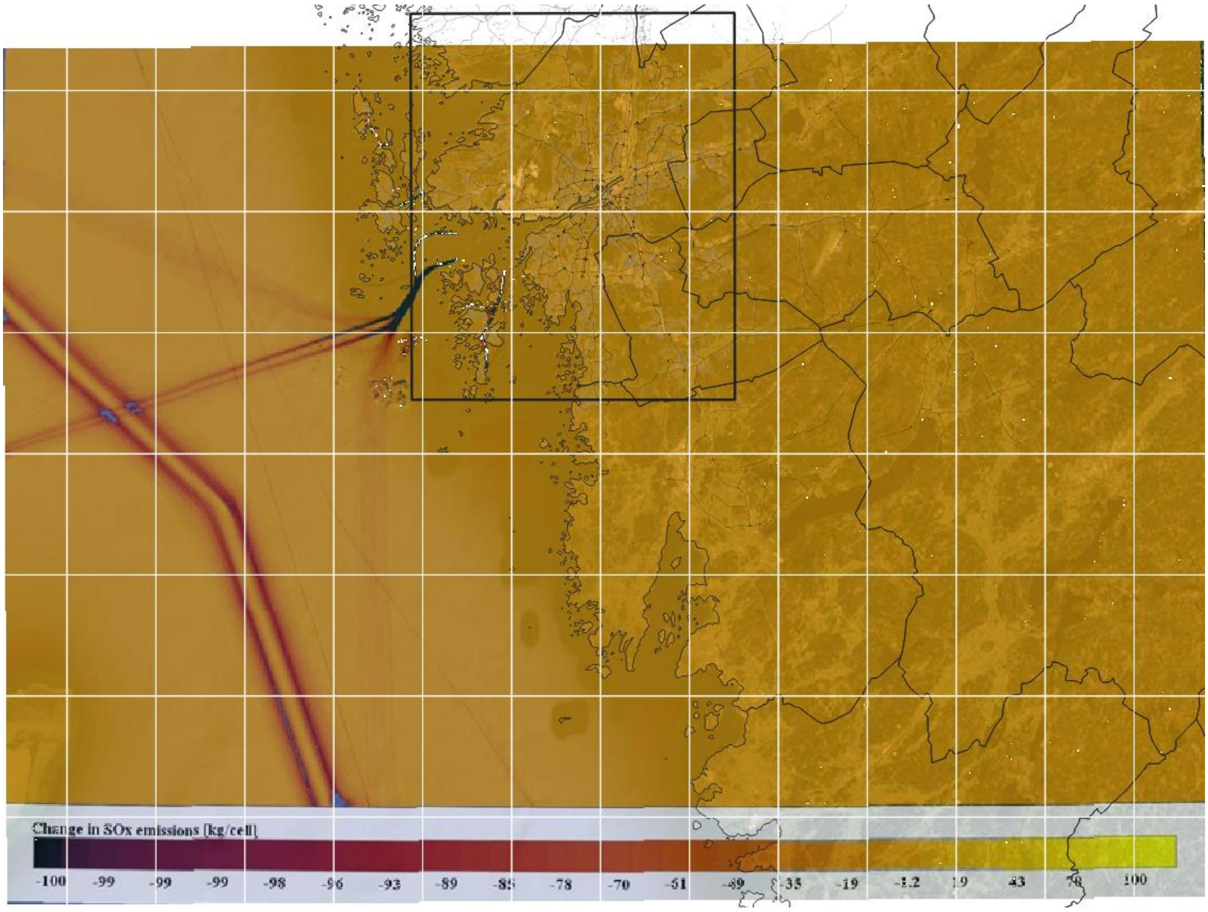

Figure S2. Difference plot of SOx emissions in 2014-2016. Note, that reduction of emissions because of policy changes happens outside the Gothenburg harbor area. In the harbor area, no major difference is found, because the requirement to use 0.1%S fuel in harbor areas has been in place since 2010. The domain indicated by this image refers to Gothenburg area referred in the above table. Image courtesy of Landsat-8 and US Geological Survey. In this figure, we have also indicated the city-scale modelling border area (the black rectangle) and the grid of the regional EMEP model (white rectangles).

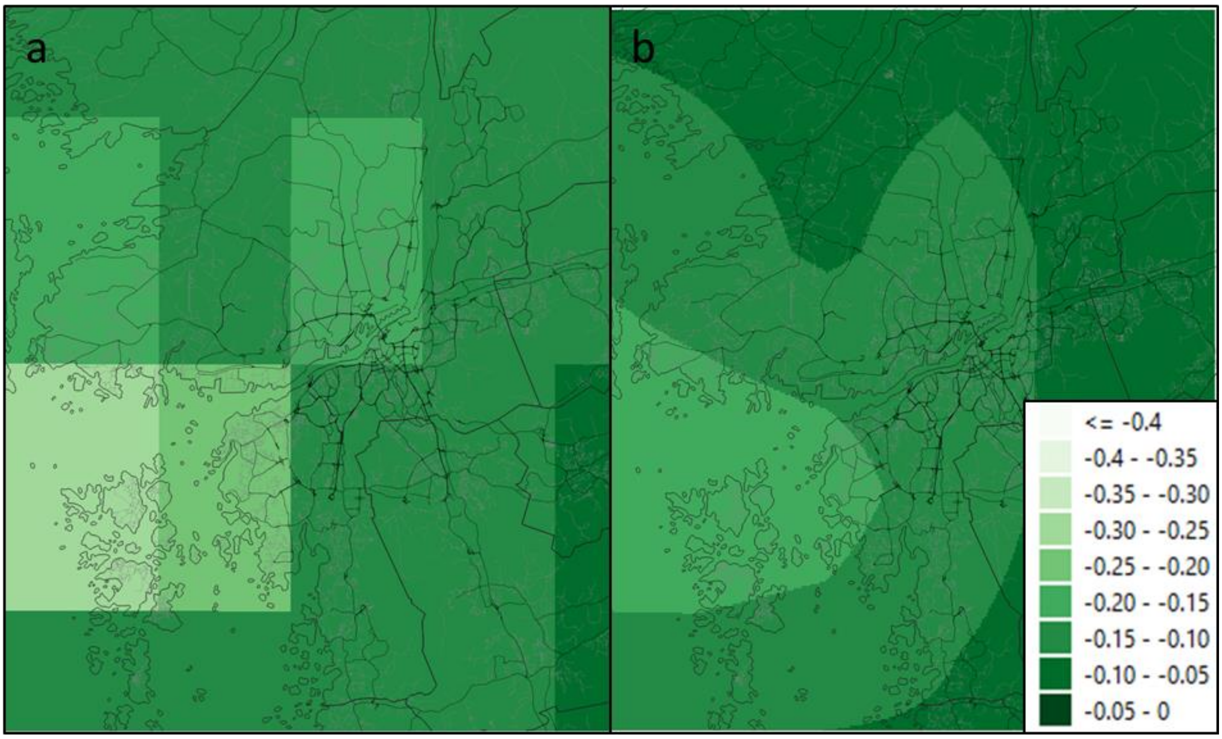

Figure S3. Comparison differences in the contributions from Baltic Sea shipping in high- and low-sulfur on $PM_{2.5}$ ($\mu g/m^3$) to Gothenburg; (a) regional model, (b) city-scale model.

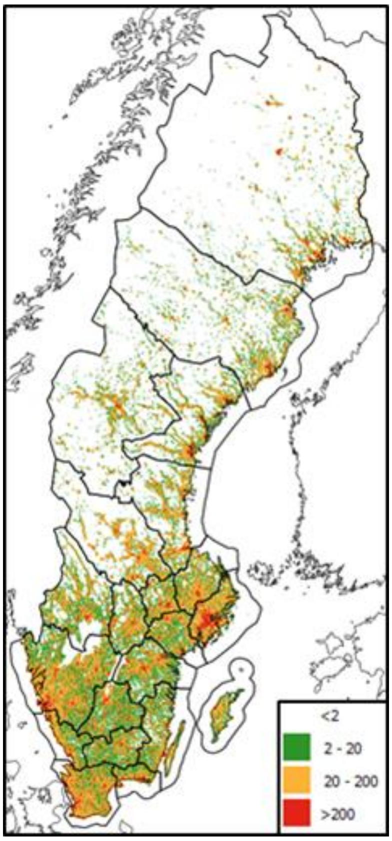

Figure S4. Population density in Sweden per km^2.

Reference

1. World Health Organization. Metrics: Disability-Adjusted Life Year (DALY). Available online: https://www.who.int/healthinfo/global_burden_disease/metrics_daly/en/2020. (accessed on 14 April 2020).
2. Salomon, J.A.; Haagsma, J.A.; Davis, A.; De Noordhout, C.M.; Polinder, S.; Havelaar, A.H.; Cassini, A.; Devleesschauwer, B.; Kretzschmar, M.; Speybroeck, N.; et al. Disability weights for the Global Burden of Disease 2013 study. *Lancet Glob. Health*. **2015**, *3*, 712–723.
3. Eriksson, C.; Bodin, T.; Selander, J. Burden of disease from road traffic and railway noise—A quantification of healthy life years lost in Sweden. Scand. *J. Work Environ. Health* **2017**, *43*, 519–525.

© 2020 by the authors. Licensee MDPI, Basel, Switzerland. This article is an open access article distributed under the terms and conditions of the Creative Commons Attribution (CC BY) license (http://creativecommons.org/licenses/by/4.0/).
